# MOBILIZING INTERDISCIPLINARY RESEARCH TO ILLUMINATE DIVERSITY ACROSS THE CANCER CARE CONTINUUM

**DOI:** 10.1093/geroni/igad104.0650

**Published:** 2023-12-21

**Authors:** Sean Halpin, Jessica Krok-Schoen

## Abstract

Cancer diagnoses increase precipitously with age, impacting diverse populations who have different experiences, needs, and resources. In our symposium, we bring together researchers from multiple disciplines to present on the diversity of older adults living with cancer and the ways that health care providers, systems, and researchers are innovating to support them. Carrion presents the knowledge of gender-specific types of cancer among older Latinos/as. Seaman presents research on the use of comprehensive geriatric assessments by oncology and primary care providers who work with older adults. Blackberry discusses the use of age-friendly models of care within cancer care. Halpin discusses the results from a scoping review of cancer therapy decision-making among patients with co-morbid dementia and cancer and their caregivers. Ultimately, we discuss the need for multiple methods to understand the experience of patients from diverse populations and with various cancer diagnoses and innovate in cancer care research.

